# Subcapsular Liver Hematoma in HELLP Syndrome: Case Report

**DOI:** 10.4021/gr2010.04.178e

**Published:** 2010-05-20

**Authors:** Murat Kapan, Mehmet Siddik Evsen, Metehan Gumus, Akin Onder, Guven Tekbas

**Affiliations:** a Department of General Surgery, Dicle University Medical Faculty, Diyarbakir, Turkey; b Department of Obstetric and Gynaecology, Dicle University Medical Faculty, Diyarbakir, Turkey; c Department of Radiology, Dicle University Medical Faculty, Diyarbakir, Turkey

**Keywords:** Subcapsular liver hematoma, HELLP syndrome, Conservative management

## Abstract

Subcapsular liver hematoma, as a rare complication of HELLP syndrome, must be managed in a tertiary center for prompt recognition and treatment with close monitoring of hemodynamic and coagulation parameters, treatment of underlying disorders, and assessment by the imaging techniques. These patients underwent different therapeutic options varying from conservative therapy to operative management, including liver transplantation. As a choice of treatment, patients with HELLP syndrome can be followed up conservatively in stable hemodynamic conditions. In this report, we presented a 32-year-old woman with subcapsular liver hematoma secondary to HELLP syndrome managed conservatively.

## Introduction

The HELLP (Hemolysis, Elevated Liver enzymes, Low Platelets) syndrome is associated with preeclampsia and known as a pregnancy-related complication. Subcapsular liver hematoma has been reported in less than 2% of pregnancies complicated by HELLP syndrome. Subcapsular liver hematoma may result in hepatic rupture. The incidence of subcapsular liver hematoma with rupture in pregnancies varies from 1/40000 to 1/250000 [1]. And this significantly increases both maternal and perinatal morbidity and mortality [2]. In this case, we report a subcapsular liver hematoma managed by conservative measures.

## Case Report

**Figure 1:**
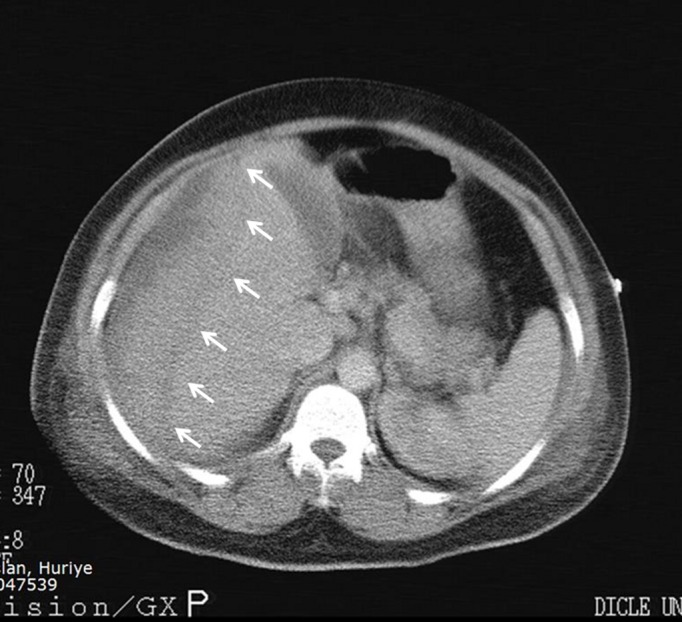
CT shows a subcapsular hematoma in the right hepatic lobe. The capsule is intact.

**Figure 2:**
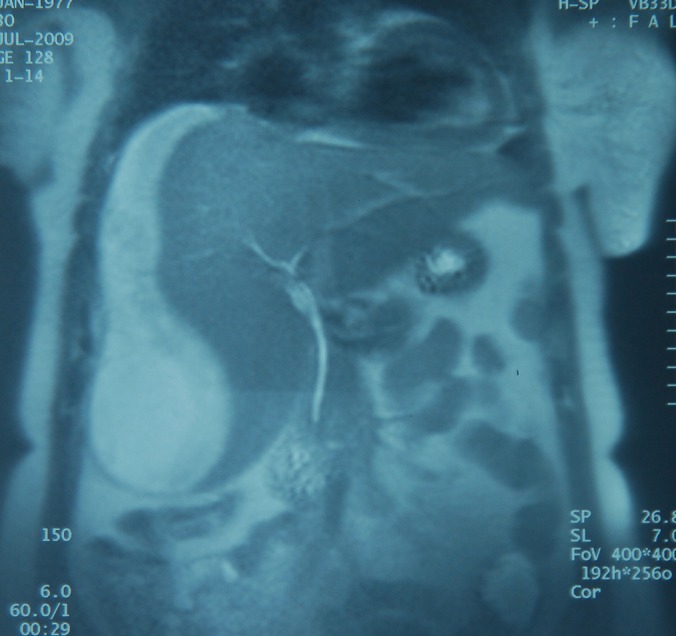
MRI shows a subcapsular liver hematoma without significant size progression at T2-weighted imaging.

A 32-year-old woman gravida 6, parity 5, was admitted at 38 weeks of gestation for delivery to a state hospital. The patient was hypertensive and had proteinuria. A cesarean section was performed because of fetal distress by Pfannenstiel incision and a 3 kilogram live-born male infant was delivered. Intravenous magnesium sulfate was given to the patient. Twenty-four hours of postpartum, the patient was transferred to Dicle University Gynecology and Obstetric department. The patient complained of increasing right upper quadrant and shoulder pain. Blood pressure measurement was 150/90 mm Hg and laboratory studies gave the following results: serum aspartate aminotransaminase (AST), 310 IU/L; serum alanine aminotransaminase (ALT), 254 IU/L; serum lactate dehydrogenase (LDH), 865 IU/L; serum creatine kinase (CK), 567 IU/L; serum urea and creatine were normal; white blood cell, 12200/mm^3^; hematocrit, 24.5%; hemoglobin, 8.6 mg/dl; platelet count, 31 x 10^3^ µ/mL. A catheterized urine specimen demonstrated proteinuria (+++). Transabdominal ultrasound (TAUS) showed a hypoechoic heterogeneous subcapsular mass, 125 x 55 mm in diameter, in anterior segment of right hepatic lobe, and also perihepatic, perisplenic, pelvic free fluid in the abdominal cavity. When diagnosis of subcapsular liver hematoma confirmed, the patient was transferred to surgical intensive care unit. After transfer to intensive care unit, a computed tomography (CT) scan was performed to patient who revealed a large well-circumscribed subcapsular liver hematoma with intact capsule in the right hepatic lobe and free fluid in abdominal cavity (Fig. 1). Patient was stable hemodynamically and no active intra-abdominal bleeding observed and she was conservatively followed up in the intensive care unit. Two units of packed red blood cells, 6 units of fresh-frozen plasma and 2 units of pooled thrombocytes transfused. Antihypertensive treatment and high dose corticosteroids had given to patient and her motion was limited. Fourth day postpartum, she suffered from shortness of breath. Thorax CT revealed a pleural effusion with a depth of 45 mm in right side and with a depth of 30 mm in left side. With diuretic treatment this problem resolved. Repeated ultrasound examinations were performed at a daily basis to monitor the results of conservative approach. On 8th day postpartum, laboratory findings were as follows: serum ALT 55 IU/L, serum AST 31 IU/L, serum LDH 267 IU/L, white blood cell 10500/mm^3^, hematocrit 32.8%, hemoglobin 11.4 mg/dl and platelet count 367 x 10^3^ µ/mL. Blood pressure measurement was 120/80 mm Hg. Follow-up magnetic resonance imaging (MRI) 12 days after referral to the intensive care unit showed constant findings without significant size progression of the hematoma with an intact liver capsule (Fig. 2). Two weeks postpartum, the patient was no complaint and discharged from the hospital in a stable status. Six weeks post partum, USG examination of patient showed no residual hematoma or free peritoneal fluid.

## Discussion

The HELLP syndrome and other hypertensive disorders are the main cause of maternal mortality [3]. The HELLP syndrome occurs in about 0.5 - 0.9% of all pregnancies and in about 10 - 20% of severe preeclampsia cases [3]. The incidence of HELLP syndrome was significantly higher in white and multiparous patients and in women with delayed diagnosis of preeclampsia and delayed delivery [4]. HELLP syndrome develops in about 70% of the cases before delivery and 30% in postpartum period [3]. A mild and self limited course to a fulminant process including multiple organ failure can be seen in HELLP syndrome. In most cases, the postpartum HELLP syndrome would resolve spontaneously within 48 hours [5]. Administration of high dose corticosteroid in postpartum period has proved to be helpful for recovery. Therefore, corticosteroid administration (10 mg of dexamethasone every 12 hours) is strongly suggested [3]. Haddad et al [6] showed that adverse maternal outcomes could be seen in 38% of women with HELLP syndrome. These major maternal complications include disseminated intravascular coagulation (DIC), abruptio placentae, acute renal failure pulmonary edema, and subcapsular liver hematoma. The liver hematoma in pregnancy was described firstly by Abercrombie [7]. Liver hemorrhage and rupture are the most eccentric and critical complications of HELLP-associated disease. Maternal mortality occurs in about 18 - 86% cases of hepatic rupture [3]. The causes of subcapsular and intraparenchymal hepatic hematomas in HELLP syndrome are not known absolutely. Liver distention and, as a consequence, right upper quadrant or epigastric pain may occur with the obstruction of blood flow in the hepatic sinusoids. Also, this obstruction may lead to periportal necrosis and, in severe cases, intrahepatic haemorrhage, subcapsular hematoma formation or hepatic rupture. A fluorescent antibody technique has been used to demonstrate fibrin deposits in the hepatic sinusoids of eclamptic patients [8].

The cases of subcapsular liver hematomas must be treated in tertiary centers for prompt recognition and optimal treatment. Because the prognosis can be changed by the timely diagnosis and treatment [1], ultrasound, CT and MRI can be used for the diagnosis [3]. Liver hematomas in pregnancy must be closely monitored by hemodynamic and coagulation parameters during the management of HELLP syndrome and other hypertensive disorders. Serial evaluation with imaging techniques, avoidance of the liver manipulation and immediately replacement of blood products are essential. Postpartum follow-up should include serial assessment with ultrasound, CT or MRI until the defect resolves [9].

Hemodynamically stable patients should be followed up conservatively. Non-operative, conservative management includes intensive medical support with infused fluid, and replacement of blood and blood products with or without percutaneous embolization of the hepatic arteries. If rupture has occurred and the patient is unstable hemodynamically, surgery can be necessary. Packing of the bleeding surfaces with drainage of the perihepatic space, packing with collagen fleece and resection are used during surgical operation. Liver transplantation has been reported when the hemorrhage cannot be controlled and acute liver failure occurs [9].

Wicke et al [1] reported a 10 year retrospective review of 5 patients with subcapsular liver hematoma. Three of them were managed conservatively and two required urgent surgical intervention, one of whom underwent liver transplantation. Kari et al [9] reported a non-surgical conservative management of hemodynamically stable patient with ruptured subcapsular liver hematoma in pregnancy. In our case, imaging techniques showed intraabdominal free fluid in the abdominal cavity. However, the patient’s hemodynamic status was stable, radiologic imaging techniques revealed an intact liver capsule and she had undergone a cesarean section before admission to our hospital at former state hospital. Therefore, we had no doubt about the risk of a serious intraabdominal bleeding and the patient didn’t undergo any surgical operation during our follow-up period.

In conclusion, we suggest that liver hematoma in pregnancy can be successfully managed without surgery if the patient’s hemodynamic status is stable and is closely monitored in a tertiary center.
